# The complete chloroplast genome of *Sparganium fallax* (Typhaceae)

**DOI:** 10.1080/23802359.2021.1917316

**Published:** 2021-06-21

**Authors:** Qiaoyu Zhang, Yi Wang, Xinwei Xu

**Affiliations:** Department of Ecology, College of Life Sciences, Wuhan University, Wuhan, China

**Keywords:** Chloroplast genome, phylogenetic analysis, *Sparganium fallax*, Typhaceae

## Abstract

*Sparganium fallax* is an aquatic perennial herb distributed in eastern Asia. The complete chloroplast genome of *S. fallax* was sequenced and assembled. The genome size was 161,838 bp in length with 36.8% GC content. Its quadripartite structure consisted of the large single copy (LSC, 89,042 bp) and small single copy (SSC, 18,774bp) regions, separated by a pair of inverted repeats (IRS) of 27,011bp. The genome contained 114 genes, including 80 protein-coding genes, 30 tRNA genes, and four rRNA genes. The phylogenetic analysis within order Poales including three *Sparganium* species showed that *Sparganium* is monophyletic and most closely related to *Typha*.

*Sparganium* L. is an aquatic perennial genus including approximately 14-19 species (Cook and Nicholls 1986, 1987; Kaul 2000; Sun and Simpson 2010). It occurs primarily in temperate and cool regions and is ecologically important in aquatic communities (Sulman et al. 2013). To date, the complete chloroplast (cp) genomes of two *Sparganium* species, *S. eurycarpum* subsp. *coreanum* (H.Lév.) C.D.K.Cook & M.S.Nicholls and *S. stoloniferum* (Buch.-Ham. ex Graebn.) Buch.-Ham. ex Juz. have been reported (Gil et al. 2019; Su et al. 2019). These two species composed one of the two clades of *Sparganium* (Sulman et al. 2013). The cp genome data of species from the other clade of *Sparganium* have not been reported. *Sparganium fallax* Graebn. is an important dominant species in highland wetlands in eastern Asian (Sun and Simpson 2010) and placed in the other clade of *Sparganium* (Sulman et al. 2013). Here, we reported complete cp genome of *S. fallax* and reconstructed phylogenetic tree for intention of confirming the monophyly of *Sparganium*.

The sample of *S. fallax* was collected from Jianning, Fujian, China (116.89°E, 26.81°N). A specimen was deposited at the herbarium of Wuhan University (www.whu.edu.cn, Xinwei Xu, xuxw@whu.edu.cn) under the voucher number Xu3372. Total genomic DNA was extracted using the DNA Secure Plant Kit (Tiangen Biotech, Beijing, China) following the manufacturer’s protocol. Library preparation and genomic sequencing on the Illumina Hiseq 2500 platform were conducted by Benagen (Benagen Inc., Wuhan, China). The obtained paired-end reads were assembled using SPAdes v.3.9.0 (Nurk et al. 2017). The assembled sequence was annotated in MPI-MP CHLOROBOX (https://chlorobox.mpimp-golm.mpg.de/geseq.html) via GeSeq (Tillich et al. 2017) with the reference cp genome of *Typha latifolia* L. (Guisinger et al. 2010; NC_013823.1), and then corrected using Geneious Prime v2020.2 (Kearse et al. 2012). Finally, the complete chloroplast genome of *S. fallax* was submitted to GenBank (Accession No. MW583115).

The chloroplast genome of *S. fallax* was 161,838 bp in length, including a large single copy (LSC) region of 89,042, a small single copy (SSC) region of 18,774 bp, and two inverted repeat (IR) regions of 27,011bp. The GC contents of LSC, SSC, IR and whole genome are 34.7%, 30.7%, 42.4%, and 36.8%, respectively. There are 114 genes annotated, including 80 protein-coding genes, 30 tRNA genes, and four rRNA genes. Of the 18 genes containing introns, three of them have two introns (*ycf3*, *clpP*, and *rps12*). Maximum likelihood analysis of Poales was conducted based on cp genomes of 13 species using RAxML v.8 (Stamatakis 2014) with 1000 bootstrap (BS) replications. The phylogenetic tree confirmed the monophyly of *Sparganium* with strong support (100% BS), which consisted of two clades including *S. fallax* and *S. eurycarpum* subsp. *coreanum* plus *S. stoloniferum*, respectively, and was most closely related to *Typha* (Figure 1).

**Figure 1.**
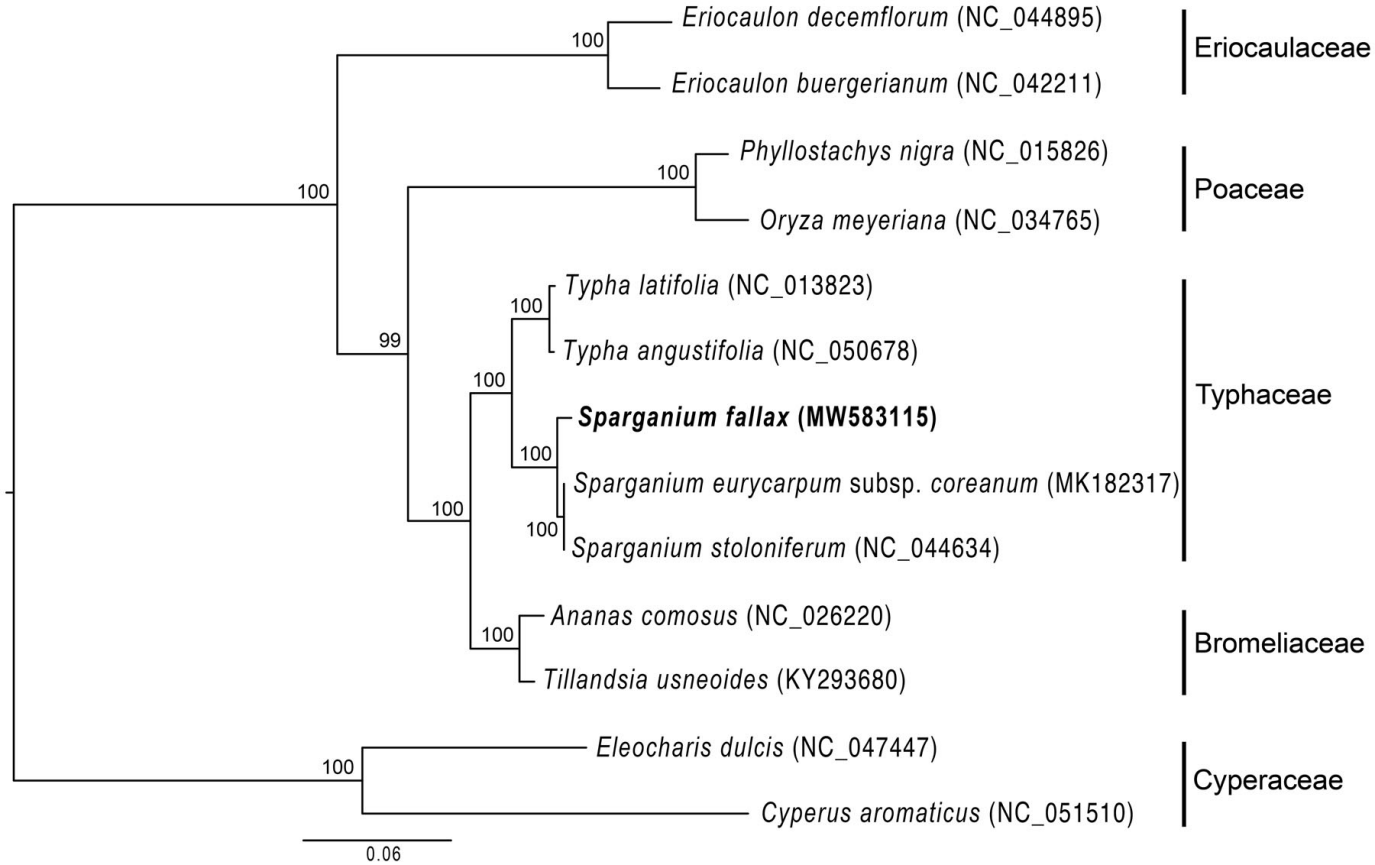
The maximum likelihood tree inferred from 13 representative chloroplast genomes of order Poales. The position of *Sparganium fallax* is highlighted in bold and numbers above each node are bootstrap support values.

## Data Availability

The genome sequence data that support the findings of this study are openly available in GenBank of NCBI at https://www.ncbi.nlm.nih.gov/ under the accession no. MW583115. The associated BioProject, SRA, and Bio-Sample numbers are PRJNA703417, SRR13684638, and SAMN17861312, respectively.
